# Pediatric Limb Asymmetry: A Unique Presentation of Angioosteohypertrophic Syndrome

**DOI:** 10.7759/cureus.62040

**Published:** 2024-06-10

**Authors:** Chaimae Salhi, Chaimae N'joumi, Youssef Banana, Adnane Benzirar, Maria Rkain, Abdeladim Babakhouya

**Affiliations:** 1 Department of Pediatrics, Mohammed VI University Hospital, Faculty of Medicine and Pharmacy, Mohammed First University, Oujda, MAR; 2 Department of Vascular Surgery, Mohammed VI University Hospital, Faculty of Medicine and Pharmacy, Mohammed First University, Oujda, MAR; 3 Pediatric Gastroenterology, Mohammed VI University Hospital, Oujda, MAR; 4 Pediatric Cardiology, Mohammed VI University Hospital, Oujda, MAR

**Keywords:** child, congenital malformation, arteriovenous fistulas, klippel-trenaunay-weber syndrome, parkes-weber syndrome

## Abstract

Parks-Weber syndrome (PWS), also known as Klippel-Trenaunay-Weber syndrome, is a rare congenital bone vascular syndrome first described in 1900. It is characterized by arteriovenous malformations in a limb, leading to disproportionate limb growth and potential heart failure. Unlike Klippel-Trenaunay syndrome, PWS manifests arteriovenous malformations with abnormal connections between the arteries and veins of the affected limb. The management of this syndrome, similar to that of Klippel-Trenaunay syndrome, relies mainly on symptomatic treatment.

We report the first case of angioosteohypertrophic syndrome diagnosed at CHU Med VI Oujda, in a patient aged seven years and eight months. This syndrome manifested primarily in the right upper limb, characterized by asymmetry in both upper limbs, thermal disparity, a cutaneous nevus, and venous ectasia in the right arm. The diagnosis was further substantiated through arteriography, confirming the presence of an arteriovenous fistula.

## Introduction

Parkes-Weber syndrome (PWS), also known as Klippel-Trenaunay-Weber Syndrome, is an exceedingly rare congenital vascular bone syndrome; it was first described in 1900 [1]. Despite the availability of various clinical series and reported cases in the literature, its etiology remains elusive [2]. It is characterized by the presence of a predominantly arteriovenous vascular malformation in a limb, resulting in disproportionate limb growth [3]. In contrast to Klippel-Trenaunay syndrome, individuals with PWS exhibit arteriovenous malformations featuring abnormal connections between the arteries and veins within the affected limb, potentially leading to cardiac insufficiency [4,5]. Management approaches, akin to those for Klippel-Trenaunay syndrome, primarily involve symptomatic treatment [1]. We present the first case of this syndrome at our institution, identified in a seven-year-and-eight-month-old male, showcasing this syndrome in the right upper limb. Our objective is to raise awareness among physicians about this condition.

## Case presentation

**Figure 1:**
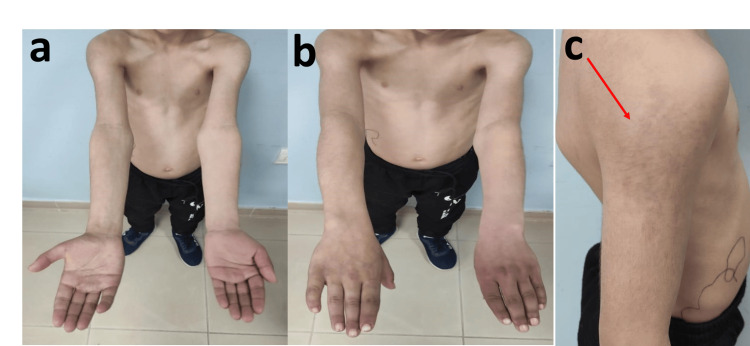
Photograph of the patient showing asymmetry of upper limbs in length and width, as well as a nevus located on the outer side of the right arm a: Anterior side. b: Posterior side. c: Cutaneous nevus indicated by the red arrow

The patient was a seven-year-and-eight-month-old male, born from a second-degree consanguineous union, with no notable medical history, particularly no history of thromboembolic disease, who was referred to our facility due to asymmetry between his upper limbs. He was the eldest of two siblings, and his brother exhibited no discernible malformations. His dominant hand was the left one. Since the age of two years, his parents had observed a temperature discrepancy between his upper limbs, alongside nevi located on the right arm's outer aspect and the dorsum of the right hand. By the age of five years, the parents had noted increasing asymmetry in the upper limbs (Figure 1).

On clinical examination, comparative measurements revealed a 3 cm elongation and slight hypertrophy of the right limb, with a 2 cm difference between the two upper limbs. The exam also showed elevated skin temperature in the right limb, a cutaneous nevus on the right arm's outer aspect and a more subtle one on the dorsum of the right hand, venous ectasia on the right shoulder's anterior aspect and the right arm, and a thrill and murmur along the humeral, axillary, and subclavian axes, indicative of an arteriovenous fistula. Associated with slight asymmetry in blood pressure measurements, a difference of 21 mmHg was observed in both systolic and diastolic blood pressures, between the right and left limbs. Other systems showed no anomalies, including no evidence of heart failure.

**Figure 2:**
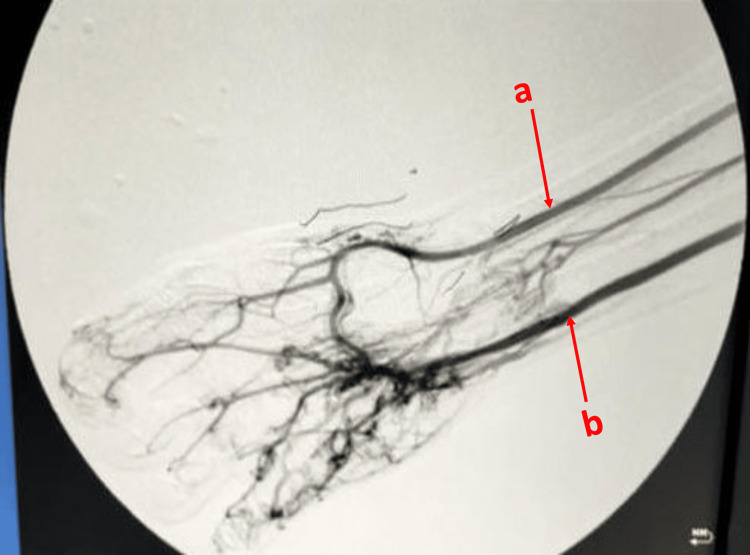
Arteriography showing the radial and ulnar arteries and the palmar arch a: Radial artery. b: Ulnar artery

**Figure 3:**
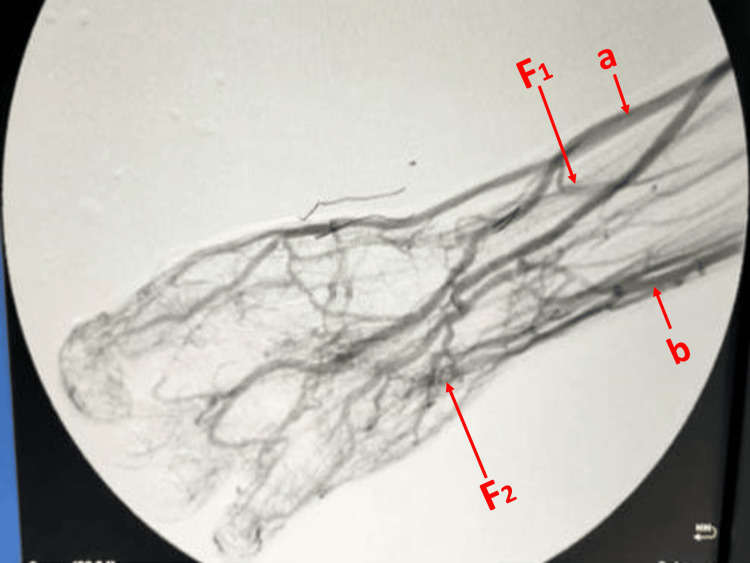
Arteriography revealing late opacification of the cephalic and ulnar veins of the forearm, as well as two arteriovenous fistulas (cephalic-radial and ulnar-ulnar) a: Cephalic vein. b: Ulnar vein. F1: Cephalic-radial fistula. F2: Ulno-ulnar fistula

Laboratory investigations and standard radiography yielded no skeletal abnormalities. Doppler ultrasound was not much contributory either. Further investigations comprised arteriography, which confirmed the presence of two arteriovenous fistulas - radiocephalic and ulno-ulnar - thus establishing the diagnosis of angioosteohypertrophic syndrome (Figures 2-3). We carried out a cardiological assessment, which revealed normal results. The patient is currently undergoing regular clinical monitoring. So far, no complications or additional symptoms have been observed.

## Discussion

PWS is a complex vascular anomaly that manifests from birth itself [6]. It is considered a rare disorder, with an estimated incidence of one case per 100,000 individuals [7]. The diagnosis of this syndrome is based on the presence of a distinctive triad comprising arteriovenous fistulas, capillary malformations (known as port-wine stains), and overgrowth of affected limbs [3]. While its exact cause remains unknown, familial cases have been reported, suggesting a pattern of autosomal dominant inheritance [2,8]. Some cases of PWS exhibit the same genetic anomaly due to a mutation in the RAS p21 protein activator 1 gene (RASA1), which affects vascular system development [9]. There is no sex or race predilection [10]. The average age of discovery is eight years, with diagnosis evident in 91% of cases at birth, and it can even be diagnosed in utero [11].

The clinical presentation varies depending on the location and extent of arteriovenous malformations [11]. The condition manifests in various ways, ranging from mild to severe forms. Mild cases commonly exhibit limb length discrepancies, increased skin temperature, dermatitis, skin discoloration, and swelling of the affected limb. Severe forms may involve additional symptoms such as pain, warmth, and pulsations in the affected limb [12]. Complications of the syndrome can include bleeding, skin infections, thromboses, and heart failure [5].

Venous malformations can occur in the superficial and deep venous systems. Superficial anomalies range from small vein ectasia to persistent embryonic veins and large venous malformations. Deep malformations encompass aneurysmal dilatation, aplasia, hypoplasia, duplications, and venous insufficiency [6]. It is noteworthy that upper limb involvement, as observed in our case, is relatively rare compared to lower limb manifestations [8]. In atypical presentations, PWS may extend beyond limb involvement to potentially affect visceral organs such as the pelvic region, and gastrointestinal tract, or, rarely, solid organs like the spleen, liver, lung, heart, kidney, or facial structures [2].

Diagnosis typically relies on clinical manifestations, while imaging is indispensable for evaluating the location and extent of arteriovenous malformations [10]. Doppler ultrasound serves as an initial tool to differentiate between high-flow and low-flow malformation, providing a foundation for further assessment with MRI, which offers detailed insights into musculoskeletal and vascular anomalies [4,8]. Moreover, this syndrome is often confused with Klippel-Trenaunay syndrome until definitive arteriovenous malformations and genetic underpinnings are elucidated [3,6].

The management entails a multidisciplinary approach, aiming to improve the quality of life through individualized treatment tailored to the patient's age and clinical profile [7]. Compression therapy constitutes a primary palliative measure for managing edema and venous ulcers, while invasive procedures such as coil embolization, stent grafting, malformation resection, or even amputation are reserved for cases with complications [11]. In the realm of medical treatment, while various pharmaceutical agents, including sirolimus, have been explored, their definitive efficacy has not been fully established [7]. Regular monitoring is essential to detect evolving complications and adjust therapeutic management accordingly [6].

## Conclusions

PWS is a rare congenital malformation requiring multidisciplinary management. Clinical assessment holds pivotal importance and guides appropriate paraclinical investigations. Doppler ultrasound serves as a key diagnostic tool, aiding in both complication screening and non-invasive lesion monitoring, often complemented by additional imaging modalities. The treatment focuses on symptom management, aiming to address both aesthetic and functional concerns while averting potential complications. Disease progression is contingent upon the extent of the lesion.

## References

[1] Girón-Vallejo O, López-Gutiérrez JC, Fernández-Pineda I (2013). Diagnosis and treatment of Parkes Weber syndrome: a review of 10 consecutive patients. Ann Vasc Surg.

[2] Weerakkody Y, Sharma R, Knipe H (2024). Klippel-Trénaunay syndrome. https://radiopaedia.org/articles/klippel-trenaunay-syndrome-1.

[3] Deshpande AA, Pandey NN, Singh SP, Kumar S (2020). Klippel-Trenaunay and Parkes-Weber syndromes: differences between congenital vascular syndromes!. Indian J Surg.

[4] Ziyeh S, Spreer J, Rössler J, Strecker R, Hochmuth A, Schumacher M, Klisch J (2004). Parkes Weber or Klippel-Trenaunay syndrome? Non-invasive diagnosis with MR projection angiography. Eur Radiol.

[5] Mahmudova MS, Shukurdjanova SM (2022). Complications of Parkes Weber syndrome. Am J Med Sci Pharma Res.

[6] Samimi M, Lorette G (2010). Klippel-Trenaunay syndrome (Article in French). Presse Med.

[7] Avilez-Soto C, Zuniga-Alfaro L (2022). Parkes Weber syndrome in a neonate: a case report. Hispanoam J Health Sci.

[8] Auzina L, Skuja E, Janis Safranovs T, Ozolins V, Kidikas H, Taurina G, Lubaua I (2020). A giant arteriovenous malformation and fistula in a newborn with Parkes Weber syndrome. Case report. Acta Med Litu.

[9] Koh HR, Lee YK, Ko SY, Shin SM, Han BH (2018). RASA1-related Parkes Weber syndrome in a neonate. Neonatal Med.

[10] Cavallaro A (2021). Aneurysms of the Popliteal Artery. Sapienza University of Rome.

[11] Banzic I, Brankovic M, Maksimović Ž, Davidović L, Marković M, Rančić Z (2017). Parkes Weber syndrome-diagnostic and management paradigms: a systematic review. Phlebology.

[12] Patel R, Durant EJ, Freed R (2021). Parkes-Weber syndrome in the emergency department. BMJ Case Rep.

